# In Vitro and In Vivo Evaluation of Magnetic Floating Dosage Form by Alternating Current Biosusceptometry

**DOI:** 10.3390/pharmaceutics16030351

**Published:** 2024-03-02

**Authors:** Gustavo Serafim Rodrigues, João Miguel Barboza, Laís Pereira Buranello, Vitor Melo Brandão, Priscileila Colerato Ferrari, Guilherme Augusto Soares, José Ricardo de Arruda Miranda

**Affiliations:** 1 Department of Biophysics and Pharmacology, Institute of Biosciences, São Paulo State University—UNESP, Botucatu 18618-689, São Paulo, Brazil; jm.barboza@unesp.br (J.M.B.); lais.buranello@unesp.br (L.P.B.); v.brandao@unesp.br (V.M.B.); guilherme.soares@unesp.br (G.A.S.); jose.r.miranda@unesp.br (J.R.d.A.M.); 2 Department of Pharmaceutical Sciences, Ponta Grossa State University, Ponta Grossa 84030-900, Paraná, Brazil; pcferrari@uepg.br

**Keywords:** ACB system, magnetic floating drug delivery systems, floating lag time (FLT), viscosity, intragastric pressure

## Abstract

Floating controlled systems seek to extend the gastric retention time (GRT) of solid pharmaceutical forms by sustaining buoyancy in the stomach without affecting gastric emptying rates. This investigation aimed to evaluate a magnetic floating drug delivery system (MFDDS) under diverse physiological conditions (pressure and viscosity) using an Alternating Current Biosusceptometry (ACB) system by conducting assessments in vitro and in vivo. For in vitro experiments, MFDDSs were placed under different pressures (760, 910, and 1060 mmHg) and viscosities (1, 50, 120, and 320 mPa·s) for evaluation of floating lag time (FLT). For in vivo experiments, eight healthy volunteers participated in two phases (fasting and fed) for gastric parameters (GRT, FLT, and OCTT—orocaecal transit time) assessment, employing the ACB system. The results indicated that pressure, viscosity, and FLT were directly proportional in the in vitro assay; in addition, increases in the OCTT (fasting = 241.9 ± 18.7; fed = 300 ± 46.4), GRT (fasting = 139.4 ± 25.3; fed = 190.2 ± 47.7), and FLT (fasting = 73.1 ± 16.9; fed = 107.5 ± 29.8) were detected in vivo. Our study emphasizes that the ACB system is a valuable technique, and it is capable of tracking and imaging MFDDS in in vitro and in vivo experiments.

## 1. Introduction

The oral route is essential in therapy as it is the most preferred and convenient route for drug delivery systems. The development of pharmaceutical forms for controlled drug release has been the target of numerous researchers over the years. Gastroretentive drug delivery systems (GRDDS) are a type of controlled drug release system [1,2,3]. The GRDDS are specially designed to increase the residence time of the dosage form in the stomach (gastric retention time, GRT). Such systems offer a substantial advantage over traditional pharmaceutical forms by ensuring a gradual release of the drug. A prolonged stay of dosage forms within the stomach, known as gastroretention, offers several therapeutic and biopharmaceutical advantages [4,5,6]. These advantages encompass enhanced drug effectiveness in the stomach, reduced variations in drug concentration plasma, increased patient adherence due to reduced dosing frequency, and improved bioavailability for specific medications that require absorption in the upper small intestine [6,7,8,9]. These systems are particularly beneficial for a range of medicines, such as those intended to act in the stomach itself, drugs with limited absorption capabilities in the upper stomach or small intestine, and substances with poor solubility in alkaline conditions (e.g., diazepam) [10,11].

Several types of GRDDS have been developed to increase the gastric retention time of pharmaceutical forms in the stomach, such as floating, bioadhesive, and expanding [12,13,14,15]. A floating drug delivery system represents a significant category of gastroretentive drug delivery mechanisms. These systems possess densities lower than gastric fluid (approximately 1.004 g/cm^3^), allowing them to float within the stomach for an extended duration without influencing gastric emptying [16,17]. They release the drug gradually while floating in the gastric contents. Their buoyancy ensures they remain distanced from the pylorus, extending their stomach stay. Consequently, these systems are primarily eliminated during the later stages of gastric emptying, resulting in an increased residence time in the stomach and enhanced regulation of fluctuations in drug plasma concentration [18,19,20,21].

However, the efficiency of gastroretentive systems in an in vivo situation is influenced by biopharmaceutical parameters and mainly by parameters related to the GIT. The biggest obstacle to gastroretentive systems is gastric motility, especially in the prandial state (fasting/fed) [22,23,24,25,26]. This state has different phases of mechanical contraction activity, with phase III being characterized by the so-called housekeeping waves [27,28]. These phases can influence the time the solid pharmaceutical form remains in the stomach, and the prandial state can affect the viscosity, density of the food bolus, and pH [29,30,31,32,33].

In addition to the physiological changes that occur in the stomach under conditions of prandial state (fasting/fed), little attention is paid to intragastric pressures and viscosity of the medium that act on gastroretentive systems [34,35,36,37,38,39]. Recent studies show that high intragastric pressures have increased the release rate of gastroretentive pharmaceutical forms [36,40,41]. This occurs primarily in the case of polymer matrix tablets and hydrogels, which are highly sensitive to these pressures, and their release rates are determined through the erosion caused by these pressures on the tablets, mainly in a prandial state (fed) [42,43]. Due to these pressures, the release rate is increased significantly, which explains irregular drug concentration profiles in plasma [27,39,44,45]. Another factor that can affect these systems is the varying viscosity of the environments in which they are placed, such as the gastric contents in the fasting and fed states [34,40,46,47,48]. The viscosity of the gastric environment can influence the floating time (or even the floating process) and, consequently, impair the drug release rate and the retention of the pharmaceutical form in the stomach [49]. Therefore, simultaneous food intake can modify the systemic availability of many medications, potentially affecting their therapeutic effectiveness, in addition to the fact that the prandial state interferes with motility.

Given that physiological factors associated with the human gastrointestinal tract substantially impact the absorption and bioavailability of orally administered drugs and considering the high costs and complexity of interpreting data from clinical trials in humans, it has become sensible to employ analytical techniques to evaluate floating systems.

Therefore, non-invasive technologies that can monitor a magnetic floating drug delivery system (MFDDS) in vitro and across GIT segments under different conditions are needed. Biomagnetic techniques to non-invasively monitor MFDDSs currently constitute a viable alternative for pharmaceutical research carried out in vitro and studies related to the physiology of the gastrointestinal tract (GIT) in humans and animals [50,51,52,53]. Alternating Current Biosusceptometry (ACB) is a biomagnetic technique used as an alternative approach in pharmaceutical research to evaluate the in vitro performance of solid dosage forms [13]. Furthermore, it is used to monitor the transit of these forms through the different segments of the gastrointestinal tract (GIT) [50,54,55]. This methodology uses ferrite powder as a tracer, an inert material, presenting stability in different pH conditions throughout the GIT, whether in acidic or basic environments [56,57]. The ACB method is based on the use of induction coils to record magnetic flux variations obtained from a magnetically susceptible material in response to the application of an alternating magnetic field [50,54]. Furthermore, it serves as a complementary tool for quality control of MDFFSs.

Previous studies have demonstrated the efficiency of the ACB system when applied in pharmaceutical research, correlatively evaluating gastric transit (GIT) and the pharmacokinetics of magnetic dosage forms (pharmacomagnetography). This methodology involves incorporating magnetic elements into pharmaceutical formulations, such as tablets or capsules, allowing real-time transit and disintegration monitoring using external magnetic sensors with imaging techniques. Thus, pharmacomagnetography is valuable for simultaneously studying drug distribution, gastrointestinal motility, and drug absorption kinetics [58,59,60].

Thus, the objective of this study was to evaluate an MFDDS in vitro and in vivo under different physiological conditions (fasting and fed states) using the ACB system.

## 2. Materials and Methods

### 2.1. Materials

The following materials were used to manufacture the floating magnetic tablets used in these studies: Ferrite powder (MnFe_2_O_4_; diameter of 90 < φ < 125 µm)—Thornton (Vinhedo, Brazil) was used as the magnetic marker; HPMC (Methocel E4M) was donated from Colorcon (Cotia, Brazil); lactose, PVP K30, magnesium stearate, and sodium starch glycolate were obtained from Henrifarma (São Paulo, Brazil); hydroxyethyl cellulose (HEC)—Colorcon (Indaiatuba, Brazil); and sodium bicarbonate—Audaz (São Paulo, Brazil). All other reagents and solvents were of analytical grade.

### 2.2. Floating Magnetic Tablets

The tablets were prepared using manganese ferrite powder (430 mg, 35.83%), magnesium stearate (18 mg, 1.5%), hydroxypropylmethylcellulose (HPMC E4M—430 mg, 35.83%), lactose (152 mg, 12.6%), polyvinylpyrrolidone (PVP K30) (10 mg, 0.83%), and sodium bicarbonate (160 mg, 13.3%). All the excipients were mixed and manually manufactured using a single punch machine (Erweka, EKOTM, Langen (Hessen), Germany) with a flat face and a 12 mm diameter at a pressure of 30 kN. Each tablet contained 1.2 g. In the present study, magnetic floating drug delivery system (MFDDS) tablet formulations were tested according to the outline in the US Pharmacopeia. Therefore, the average weight, hardness, and friability were assessed for all tablet formulations.

In the study, solutions were prepared using a specific method to simulate gastric fluid and create solutions with varying viscosities. The following steps were followed: A simulated gastric fluid was designed according to USP 23 with (SGF) and without pepsin (SGF). The solution consisted of 900 mL of distilled water and 1.6 mL of 0.1 N HCl and was checked on a calibrated pH meter (Hanna pH 210, Smithfield, VA, USA) until it reached pH 1.2. The solution was maintained at 37 ± 0.5 °C for 30 min in a water bath to replicate body temperature.

To create solutions with different viscosities, a beaker containing 330 mL of distilled water was preheated in a water bath at 80 °C. After performing the preliminary screening study, hydroxypropylmethylcellulose (Methocel K4M, Colorcon Co., Dartford, UK) was selected as a chemically inert, water-soluble, relatively readily dispersible non-ionic polymer aimed to mimic the effect of increased viscosity. Once the desired temperature (80 °C) was reached, varying amounts of the polymer HEC (2, 4, and 6 g) were weighed and added to the solution. The solution was stirred at 75 rpm using a magnetic stirrer for 30 min until it cooled to room temperature. Subsequently, 670 mL of distilled water was added, totaling 1 L of solution. This solution was continuously agitated for 12 h (overnight). The temperature of 80 °C was maintained to ensure complete dissolution of the polymer particles. After 12 h, the pH of the solutions was measured.

These procedures were carried out to create solutions with different concentrations of HEC polymer and varying viscosities while replicating gastric and body temperature conditions. These solutions were employed in the study to evaluate various parameters of interest.

### 2.3. Rheological Measurements

Rheological measurements were performed using the Brookfield RV viscometer (Waters Technologies—São Paulo, Brazil). The spindle used was the SC4-14, which had a 40 mm diameter, an angle of 1°, and a spindle-to-sample gap that varied from 0.1 to 80 Pa·s. The initial rotational speed of the viscometer was set at 1 rpm and was gradually increased until reaching a final speed of 170 rpm. Data were recorded at 20 different rotational speeds, and each data point had a recording time of two minutes. The shear rate ranged from 0.05 s^−1^ to 80 s^−1^, covering a wide range of flow conditions. Media samples were preheated at 37 °C. All measurements were performed in triplicate.

### 2.4. Floating Assessment and Magnetic Method by ACB

In vitro measurements and exploratory procedures were conducted to assess the floatability of the proposed formulation for subsequent project phases. Three random tablets were selected from the batch of prepared tablets. A standard acidic solution, simulating the intragastric fluid (SGF), was prepared with a nearly constant viscosity of approximately 1 mPa·s. The SGF consisted of 900 mL of distilled water and 1.6 mL of 0.1 N HCL, achieving a pH of 1.2. The SGF was maintained at 37 ± 0.5 °C in a water bath for 30 min to ensure stability. Each tablet was individually placed into the SGF, and its buoyant properties were visually inspected. Two critical parameters were recorded: floating lag time (FLT) and total floating time (TFT).

Magnetic measurements were conducted using an automated ACB sensor. The MFDDSs were placed within a dedicated container containing 900 mL of a 0.1 N HCl solution at pH 1.2 to carry out this experiment. The ACB was affixed to support and positioned on a computerized XYZ table in front of the container. A software routine was developed to control scanning position and timing using Mach3 Professional 2.0 software (ArtSoft, Fayette, ME, USA). Data acquisition was executed by altering the sensor’s position within a 13 × 13 cm grid marked on the container, with increments of 5 mm. Signals were obtained through an analog–digital board (NI PCI-6030E, National Instruments Inc., Austin, TX, USA) and LabView 2010 v 10.0 software (National Instruments Inc.). Image processing was carried out in the Python environment, and vertical fluctuation motion was observable, enabling the determination of the MFDDS floating lag time (FLT). Automated magnetic measurements were performed using ACB over 8 h. The area of the magnetic tablet was calculated through these scans.

### 2.5. In Vitro Studies

Studies on the floating lag time (FLT) of MFDDSs were conducted using a specifically designed testing apparatus, allowing for the control of medium pressure. The testing apparatus comprised a sealed 2 L Erlenmeyer flask fitted with a stopper and equipped with a manometer to control pressure conditions (magnetic stir bar, 50 rpm, 37 ± 0.5 °C). Simulated gastric fluids (pH = 1.2; 0.1 N HCl) with different viscosities (1, 50, 120, and 320 mPa·s) were used for FLT testing [33,47]. Based on previous studies [41], pressure values within the range found in the stomach were established for evaluation. Different pressures, including atmospheric pressure, an additional 150 mmHg, and another 300 mmHg, were applied to the testing apparatus to mimic the behavior of the stomach interior, characterized by gastric tone oscillation influenced by prandial state and compliance [40,61].

After the simulated gastric fluid and magnetic stir bar were added to the apparatus, a magnetic stirrer was used for rotation and temperature control of the medium. Following this procedure, the MFDDSs were inserted into the apparatus and the system was sealed. With the manometer attached, different pressures used in the study were applied. Tests were performed in triplicate for each applied pressure and different viscosity value, with the aim of evaluating the FLT.

### 2.6. In Vivo Study Protocol

**Figure 1:**
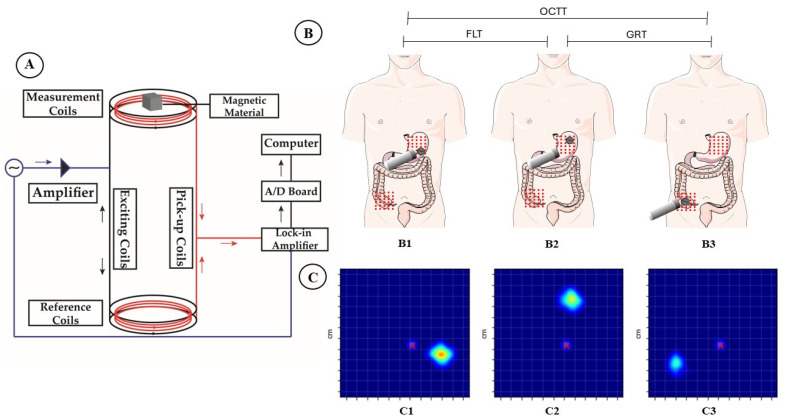
Schematic diagram of the in vivo study methodology. (**A**) Schematic representation of the Alternating Current Biosusceptometry (ACB) system. The red lines indicate the pickup coils, and the black lines indicate the excitation coils. (**B**) Region of the stomach and colon monitored by the ACB mono-channel system at three different moments: (**B1**) ingestion of the magnetic tablet; (**B2**) start of floating of the magnetic tablet; and (**B3**) arrival of the tablet in the colon. (**C**) Reconstructed image of the magnetic tablet at three different moments: (**C1**) ingestion of the magnetic tablet; (**C2**) start of floating of the magnetic tablet; and (**C3**) arrival of the tablet in the colon. Red dots represent each position measured on the abdominal surface, and red x represents the umbilical scar.

Eight healthy volunteers (both sexes, aged between 18 and 30 years, body weight between 50 and 80 kg, and BMI < 22 kg/m^2^) were enrolled. Exclusion criteria included regular use of drugs that may interfere with gastrointestinal motility or pH, like antiemetics, prokinetics, antibiotics, opioids, laxatives, and macrolides, pregnancy, smoking, abdominal surgery, chronic diseases, and any other disorders that may affect GI motility. Volunteers passed through an initial phase for gastric projection identification, which consisted of scanning all gastric projections with a mono-channel ACB sensor until finding the high magnetic signal intensity point (stomach). This point was marked with x-y coordinates, setting the reference on the umbilical scar. The study covered two moments (prandial state) of GI motility: fasting and feeding, as it was a randomized and double-anonymized comparative study to evaluate the magnetic behavior of floating tablets. There was a 7-day period separating fasting and fed procedures for the same subject. After 8 h of overnight fasting, a magnetic floating tablet was offered to all volunteers with 200 mL of Del Valle^®^ orange juice (fasting and fed states). In fed-state measurements, 30 min before taking the magnetic tablet, the volunteer received a standard meal consisting of 2 slices of Pullman^®^ loaf bread and a slice of ham and mozzarella. The single-channel ACB sensor is based on a double magnetic flux transformer, with a pair of exciter/detector coils acting as reference and another as measurement, excited by a voltage of 20 V and a frequency of 10 kHz, with the output signal detected in a lock-in amplifier. The ACB sensor was positioned over the gastric projection region, performing continuous monitoring until the tablet fluctuated and reached the cecum (Figure 1). At the end of the procedure, volunteers received a standard lunch (Perdigão^®^ lasagna with Del Valle^®^ orange juice, Perdigão, Brazil).

Floating lag time (FLT), gastric retention time (GRT), and orocaecal transit time (OCTT) were determined by scanning gastric projections in different regions [58,60]. By collecting data on magnetic field deformation intensity over time and registering it in a data matrix, images corresponding to the tablet’s position at specific times can be obtained.

This study was approved according to the Ethics in Research of Botucatu Medicine School, São Paulo State University (UNESP) protocol and under the Declaration of Helsinki. All volunteers signed the Informed Consent Form before they participated in the study (Certificate of Presentation for Ethical Appreciation: 49323221.3.0000.5411).

### 2.7. Statistical Analysis

The experiments to evaluate the floating lag time (FLT) for different pressures and viscosities were represented using mean ± standard deviation. Gastrointestinal transit parameters were analyzed using a paired Student’s *t*-test. We perform normality tests and ANOVA for multiple comparisons, revealing significant differences between the means of all parameters analyzed in the study. As a result, we performed the Tukey test to identify specific groups that presented statistical variations from each other. Magnetic signals were analyzed utilizing MatLab (MathWorks, Natick, MA, USA) and Origin software (Version 2016, OriginLab Corporation, Northampton, MA, USA). Data analysis and statistical procedures employed GraphPad Prism v 8.0.1 (GraphPad Software, La Jolla, CA, USA). Differences were considered significant when *p* < 0.05.

## 3. Results

### 3.1. In Vitro Studies

The pharmacotechnical evaluation of floating magnetic tablets was carried out as a primary test regarding the FLT and TFT parameters. The results were 5 ± 0.86 min and >24 h, respectively. This TFT demonstrates that the matrix tablets achieved stable fluctuation performance for a long time.

**Figure 2:**
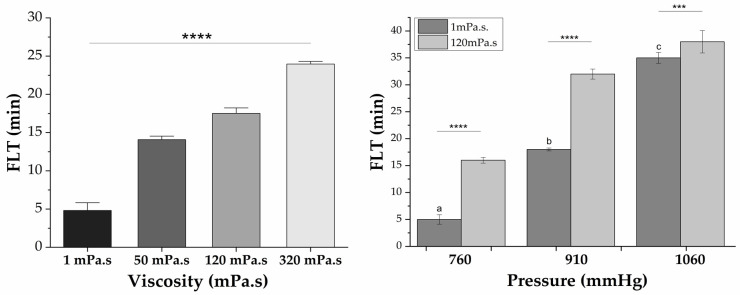
Magnetic image of the MDFFS before and after swelling and floating ((a), (b)). In (c), magnetic area variation over time graphic. *** *p* < 0.001, **** *p* < 0.0001.

To evaluate the swelling of the tablets, an experiment was carried out involving scanning the tablets at different times using the single-channel ACB system. Figure 2 shows examples of the images obtained in this experiment and the results of the tablet’s magnetic area quantification over time. This figure shows the image of a tablet immediately after being added to a specific liquid container (initial magnetic area—first point on the graph, shown in Figure 2 (a). Figure 2 (b) is the magnetic representation of the tablet after 8 h in a liquid medium. It can be noticed that the swell and fluctuation processes occurred, and the tablet is more significant in area than the initial one. Figure 2 (c) presents a graph of variation of the quantified magnetic area of the images obtained.

The FLT was 6 ± 0.8 min, as illustrated in Figure 2 (c); an average exponential fitting provides a half-time (T_1/2_—half the increase in area) of around 45 ± 7 min. The arrow indicates the FLT for this measurement.

**Figure 3:**
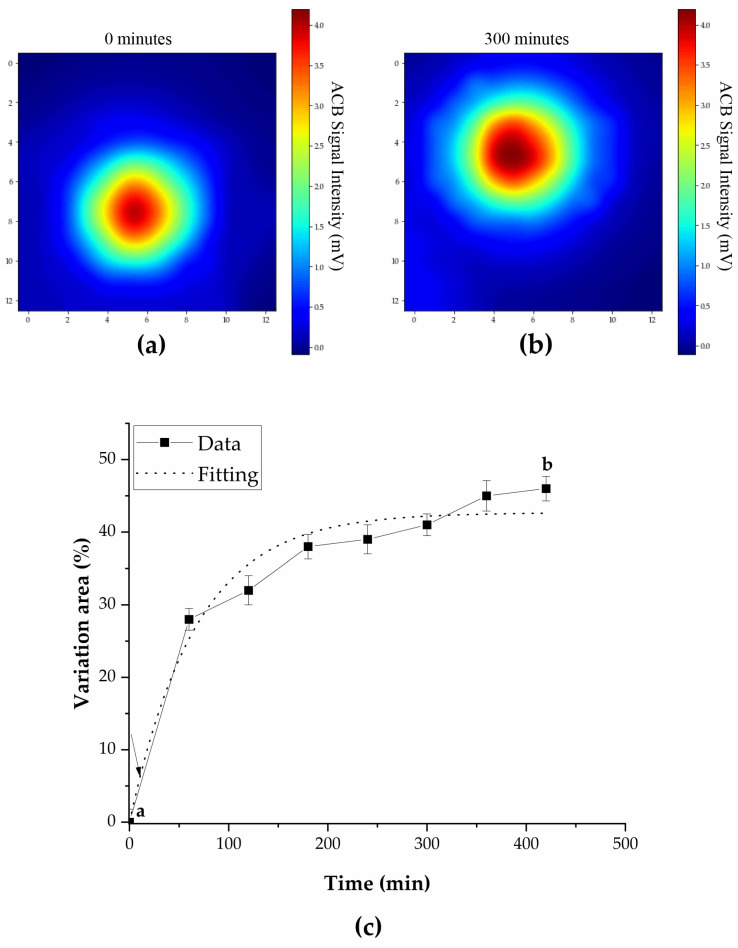
FLT of MFDDSs for different pressures (760, 910, and 1060 mmHg) and viscosities (1 and 120 mPa·s). Different letters indicate statistical differences between groups (*p* < 0.05). (**a**) First imaging, before fluctuation, right after positioning the tablet in the recipient; (**b**) Second imaging, after tablet’s fluctuation; (**c**) Magnetic area increase of magnetic floating dosage form over time.

**Figure 4:**
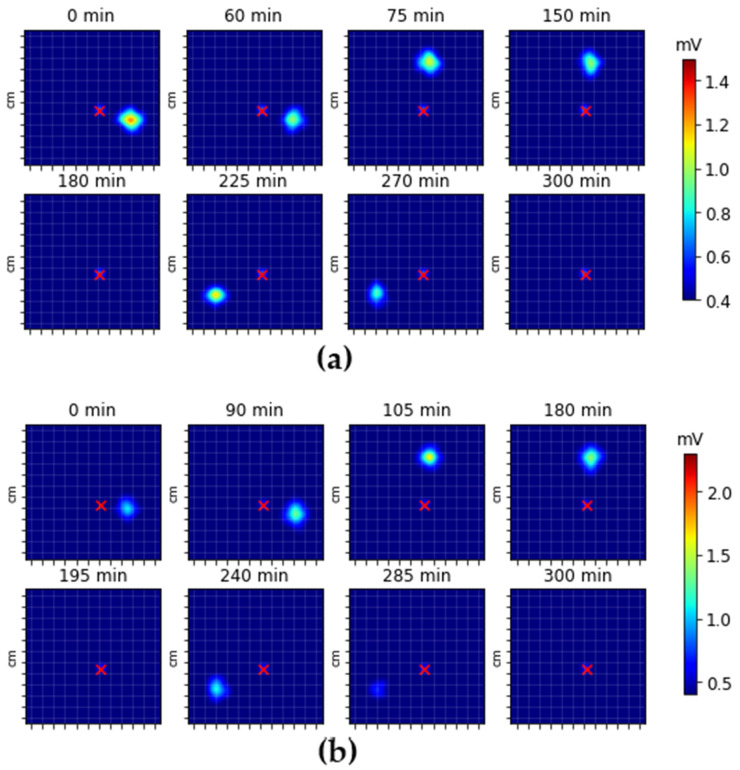
Magnetic real-time tracking, monitoring, and imaging of MFDDSs in vivo in two prandial states: fasting (**a**) and fed (**b**). The red cross indicates umbilical scar reference.

Figure 3a shows the means and standard deviations (SDs) of the FLT for the different viscosities applied in the study (1, 50, 120, and 320 mPa·s) with pressure maintained at 760 mmHg. Figure 3b shows the different pressures applied in the study (760, 910, and 1060 mmHg), with viscosity held at 1 and 120 mPa·s. The FLT values in Figure 4a were 4.46 ± 0.88, 14.4 ± 0.65, 17.2 ± 0.61, and 24.1 ± 0.43. The FLT values for the medium with a viscosity of 1 mPa·s when applying the different pressures were 5 ± 0.88 min, 18 ± 0.27 min, and 35 ± 1.0 min.

Comparing the times obtained at the same pressure, the increase in FLT differs with varying viscosity. In a possible situation close to the real one (viscosity of 120 mPa·s and pressure of 910 mmHg), the FLT value was 32 ± 0.93 min, 14 min more than in the situation with a viscosity of 1 mPa·s and pressure of 910 mmHg. For pressures of 760 and 1060 mmHg, the FLT was 16 ± 0.55 min and 38 ± 2.07 min, respectively.

The increase in FLT is observed with an increase in pressure, demonstrating the influence of pressure on the time taken for the tablet to float after its insertion into the medium.

### 3.2. In Vivo Studies

Figure 4 shows sequential images of the fasting (Figure 4a) and fed (Figure 4b) states measured with volunteers. It is noticed that the FLT, GRT, and OCTT were modified due to the prandial state.

Table 1 illustrates the data obtained for eight healthy volunteers on gastrointestinal transit parameters (GRT and OCTT) and the FLT parameter evaluated in both prandial states (fasting and fed). The fasting group presented lower indices of the parameters assessed concerning the fed group. All results are presented as mean and standard deviation (SD).

This reinforces that in all measurements with volunteers, the floating state of the MFDDS was maintained, indicating that they presented good conditions for maintaining their purpose of floating. After FLT, the tablets continued to fluctuate until the complete retention time.

## 4. Discussion

Gastroretentive drug delivery systems (GRDDS) are ingeniously engineered to extend their residence within the stomach. This prolongation leads to an extended window during which drugs can be effectively absorbed, enhancing their bioavailability [62]. These systems offer several advantages for a spectrum of medications. For instance, drugs intended to act directly in the stomach, such as the antibiotic metronidazole used for *Helicobacter pylori* eradication [63] and those with limited absorption in the upper stomach or small intestine, like simvastatin [64] and norfloxacin [65]. Moreover, GRDDS are an optimal choice for drugs that are prone to degradation in the intestinal or colonic environments, such as captopril [66] and for substances with poor solubility under alkaline conditions, like diazepam [67]. Various strategies have been proposed to achieve gastric retention and prevent unpredictable dosages from emptying. These may involve the co-administration of drugs or pharmaceutical additives that modulate gastric motility patterns, thereby delaying gastric emptying.

Nevertheless, the study of the behavior of these GRDDS is essential given the possibility of influence of pressure, viscosity, and prandial state. Furthermore, in vivo measurements configure a realistic situation of these formulations’ behavior in the GIT.

One cost-effective technique for assessing formulation behavior in the GIT is the ACB system, which is sensitive and does not require electromagnetic shields. Studies have shown that the ACB system can determine the onset of the disintegration process of magnetic tablets coated with Eudragit E100 and under the influence of omeprazole by acquiring images of the volunteer’s stomach at different times. In another study, simultaneous image acquisition and calculation of the area increase made it possible to observe the difference in the disintegration process between volunteers who received placebo tablets and those who received prucalopride. Therefore, the system effectively monitors and locates magnetic tablets, enabling the delimitation and understanding of the processes that occur in the GIT. In this study, we aimed to obtain in vitro behavior of an MFDDS (swelling and floating lag time) in the face of different pressures and viscosities and in vivo (floating lag time, gastric retention time, and orocaecal transit time) in the fasting and fed state.

Figure 2 illustrates the behavior of the increase in area of the formulation due to the swelling process and concomitant water uptake. We quantified the average T(1/2) values, in which we considered time to reach 63% stabilization in the magnetic area increase curve with an average and standard deviation of 170 ± 15, indicating an extended time window of formulation dynamics. It is important to note that Corá et al. found a direct relationship between an increase in surface area and dissolution while using a different type of magnetic solid dosage form [68]. These measurements provide further evidence of the reliability of the biomagnetic technique in assessing the changes over time in the magnetic field distribution sensor (MFDDS) area. The effectiveness of MFDDS is affected by pressure and viscosity; by evaluating TFT and FLT in different viscosity/pressure combinations and under controlled situations (water and ambient pressure), the impact of these factors can be better understood.

Figure 3a illustrates a tablet’s FLT concerning the medium’s viscosity. A significant positive correlation between the medium’s viscosity and FLT is evident, indicating that as viscosity increases, so does the time to start the floating process (FLT). This phenomenon can be attributed to the increased resistance that a more viscous medium offers to the downward movement of the tablet, thereby prolonging its FLT. In addition, the increase in FLT is likely due to the differing ionic strength values (0.4 M versus 0.18 M) presented by the media with viscosities of 1 mPa·s and 120 mPa·s, respectively. This difference in ionic strength results in a decreased water permeation rate through the tablet, consequently leading to a higher FLT [69].

Figure 3b presents an examination of the floating lag time (FLT) of a tablet in two different medium viscosities (1 and 120 mPa·s) under three distinct pressures (760, 910, and 1060 mmHg). The results depicted a clear trend that the FLT increases with pressure, a phenomenon more pronounced for the medium with a viscosity of 120 mPa·s. The FLT difference between the two viscosities is particularly noticeable at the lowest pressure (760 mmHg), where the FLT for 120 mPa·s is significantly higher. As the pressure increases, the difference in the tablet’s floating ability becomes smaller but remains significant. This can be seen as a connection between the viscosity and pressure of the medium and the tablet’s floating behavior. When pressure increases, the tablet may float better in more viscous media. It is possible that the physical properties of the medium and the tablet change under high-pressure conditions. These insights suggest that both the viscosity of the medium and the pressure play pivotal roles in determining a tablet’s FLT. Therefore, meticulous consideration of these factors is necessary when designing and optimizing tablet formulations to ensure adequate flotation and, consequently, controlled drug release.

In this study, the profile of MFDDS in the gastrointestinal tract (GIT) was assessed through in vivo acquired images. Figure 3b showed a significant increase in FLT (floating lag time) in the range of 50 to 120 mPa·s, which is more representative of real situations [49]. This increase may cause rapid emptying of the floating tablet into the duodenum, preventing it from achieving its greater purpose of retention and prolonged release. The significant increase in FLT is because the more viscous medium slows down the diffusion of water into the tablet, which delays the beginning of swelling and gel formation, which reduces the tablet’s density [70,71]. In addition, the tablet’s ability to react with gastric acid and produce carbon dioxide is slowed down by the food in the stomach. As a result, the time it takes for the tablet to dissolve varies greatly, depending on the type of food consumed, especially during the postprandial state.

Table 1 shows the GRT, OCTT, and FLT parameters obtained after processing the in vivo images. It was observed that the fasting group exhibited lower GRT, OCTT, and FLT values compared to the fed group. It is important to highlight that, under fasting conditions, the viscosity in the stomach remains at its basal state of 1 mPa·s, indicating an accelerated contractile activity in the stomach and, consequently, the potential for rapid tablet emptying [45,72].

In the fed group, the increase in the analyzed parameters is associated with the elevated viscosity in the stomach after the ingestion of food, related to an increase in intragastric pressure. The influence on the viscosity of the medium leads to interactions between the various excipients in the tablet formulation and the food bolus (proteins, fat, sugar), forming a hydrophobic barrier arounsd the tablet. This barrier affects the diffusion rate of gastric fluid into the tablet, causing a delay in the formation of the gelled layer and the release of carbon dioxide and, consequently, a delay in FLT, GRT, and OCTT [69].

## 5. Conclusions

Gastroretentive drug delivery systems, specifically floating systems, are an attractive solid pharmaceutical form because they can float and extend the time drugs stay in the stomach, allowing for controlled and site-specific drug delivery.

Using the ACB technique, it was possible to monitor MFDDSs in vitro and inside the GIT. The impact of pressure and viscosity on MFDDSs in vitro was evaluated. The results showed a significant direct relationship between pressure, viscosity, and MFDDS.

In terms of in vivo measurements, it was observed that the prandial state affects fluctuation and physiological parameters such as gastric retention time (GRT) and overall transit time (OCTT), significantly delaying them.

The ACB system has demonstrated its effectiveness in real-time monitoring, tracking, and imaging of MFDDSs, thereby establishing itself as a valuable technique for evaluating the behavior of floating delivery systems in the gastrointestinal tract. This may be useful in developing new drug delivery systems that can effectively target specific areas of the gastrointestinal tract, improving the efficiency of drug delivery outcomes.

## Figures and Tables

**Table 1:** Comparison of gastrointestinal transit parameters in the group of fasting and fed volunteers. Different letters indicate statistical differences between groups (*p* < 0.05).

	Fasting			Fed		
Subjects	GRT (min)	OCTT (min)	FLT (min)	GRT (min)	OCTT (min)	FLT (min)
1	120	240	60	180	300	90
2	150	225	75	180	240	105
3	100	225	60	135	270	75
4	150	270	90	225	330	120
5	120	225	75	285	340	165
6	135	240	90	150	240	105
7	175	240	90	205	365	125
8	165	270	45	165	315	75
Mean	139.4 ^a^	241.9 ^a^	73.1 ^a^	190.2 ^b^	300 ^b^	107.5 ^b^
SD	25.3	18.7	16.9	47.7	46.4	29.8

## Data Availability

Almost all data are presented within the manuscript (figures and tables). The raw data presented in this study are available on request from the corresponding author.
